# Accelerometry-enabled measurement of walking performance with a robotic exoskeleton: a pilot study

**DOI:** 10.1186/s12984-016-0142-9

**Published:** 2016-03-31

**Authors:** Luca Lonini, Nicholas Shawen, Kathleen Scanlan, William Z. Rymer, Konrad P. Kording, Arun Jayaraman

**Affiliations:** Max Nader Lab for Rehabilitation Technologies and Outcomes Research, Rehabilitation Institute of Chicago, 345 E Superior St, Chicago, IL 60611 USA; Department of Physical Medicine and Rehabilitation, Northwestern University, Chicago, IL 60611 USA; Sensory Motor Performance Program, Rehabilitation Institute of Chicago, Chicago, IL 60611 USA

**Keywords:** Spinal cord injury (SCI), Lower limb exoskeleton, Outcome measure, Walking skills, Paraplegia, Wearable accelerometer, Naive Bayes

## Abstract

**Background:**

Clinical scores for evaluating walking skills with lower limb exoskeletons are often based on a single variable, such as distance walked or speed, even in cases where a host of features are measured. We investigated how to combine multiple features such that the resulting score has high discriminatory power, in particular with few patients. A new score is introduced that allows quantifying the walking ability of patients with spinal cord injury when using a powered exoskeleton.

**Methods:**

Four spinal cord injury patients were trained to walk over ground with the ReWalk™ exoskeleton. Body accelerations during use of the device were recorded by a wearable accelerometer and 4 features to evaluate walking skills were computed. The new score is the Gaussian naïve Bayes surprise, which evaluates patients relative to the features’ distribution measured in 7 expert users of the ReWalk™. We compared our score based on all the features with a standard outcome measure, which is based on number of steps only.

**Results:**

All 4 patients improved over the course of training, as their scores trended towards the expert users’ scores. The combined score (Gaussian naïve surprise) was considerably more discriminative than the one using only walked distance (steps). At the end of training, 3 out of 4 patients were significantly different from the experts, according to the combined score (*p* < .001, Wilcoxon Signed-Rank Test). In contrast, all but one patient were scored as experts when number of steps was the only feature.

**Conclusion:**

Integrating multiple features could provide a more robust metric to measure patients’ skills while they learn to walk with a robotic exoskeleton. Testing this approach with other features and more subjects remains as future work.

## Background

Clinical scores of walking ability are crucial in many areas of physical rehabilitation to assess the efficacy of a therapeutic intervention or an assistive device, as well as to discriminate the ability between different patients [1, 2]. One domain of interest is evaluating functional ambulation in individuals who suffered a spinal cord injury (SCI). Even though many outcome measures target the SCI population [3, 4], currently there exist no specific measures targeting the ability of a patient to use a lower limb robotic exoskeleton to walk overground and achieve functional ambulation.

Lower limb exoskeletons are bilateral powered orthoses designed to provide assistance for sit-to-stand and for walking and, in some cases, to assist lower extremity function in individuals with incomplete or complete SCI [5–8]. Currently, several exoskeletons are transitioning from purely research and rehabilitation devices to personal mobility systems that individuals with SCI could use to walk inside their home and in their communities [9, 10]. A paradigmatic case is the ReWalk™, which has been approved by the Food and Drug Administration to be sold to individuals with SCI as a take-home personal mobility device.

Quantitative clinical assessment of exoskeletons is fundamental to evaluate their safety and effectiveness when used by individuals with disabilities. Specifically, individuals with complete SCI, who aim at taking an exoskeleton home as a personal mobility device, require an intensive training protocol to become independent users. Such training is typically delivered in a clinical setting and therefore clinicians need a robust metric to evaluate if a patient has reached a level of ability and expertise to independently use the device at home and in the community. Obtaining a robust index of the patients’ walking skills with an exoskeleton could also be used to inform health insurance companies about the actual improvements in functional mobility for potential reimbursement. This point is crucial as the cost of these devices is extremely high and therefore any support funding has to be justified.

The primary clinical outcome measures currently used to assess functional ambulation with exoskeletons are the 6-Minute-Walk-Test (6MWT) and the Ten-Meter-Walk-Test (10mWT) [11, 12]. These two tests measure, respectively, the distance walked in six minutes and the time to walk over a distance of 10 m, while walking at a constant speed. Despite being validated in spinal cord injury populations [13], it is questionable whether these measures are sufficient to fully evaluate a patient skill and the device efficiency. Indeed, other studies have measured additional features to characterize walking skills with robotic exoskeletons.

Specifically, amongst the features quantified there are: the kinematics of the hip, knee and ankle joints in patients trained to use the ReWalk™ [14], as recorded via a motion capture system; the exertion level based on the heart rate normalized to the walking speed (i.e. physiological cost index) [15] and the oxygen uptake [16, 17]. Other metrics used include the variation in vertical and lateral amplitude of the head motion [18], ground reaction forces analysis [19] and the ability to maintain eye contact to assess cognitive effort [20]. Even when multiple features were measured, each study reports the values of each feature individually to characterize functional ambulation with exoskeletons. Therefore it is unclear how each feature contributes to the overall expertise of a subject. Furthermore, some of the captured features require complex and expensive lab equipment, commonly seen only in large hospitals and university settings.

In the current study, we propose to combine multiple features of walking performance by estimating their probability distribution over a set of expert users who have been previously trained extensively to use the exoskeleton. New participants are then scored based on how well their features fit the experts’ probability distribution. Building on this principle, we define a new score to quantify walking ability with exoskeletons: the *Gaussian Naïve Bayes surprise*. The term surprise is derived from information theory and represents the amount of unexpected information provided by an event [21]. We apply our score to quantify the walking skills of four individuals with complete SCI, as they are trained to use the ReWalk™ exoskeleton. Four features are computed from the trunk accelerations, which are recorded using a commercial wearable accelerometer while subjects perform a 6MWT with the exoskeleton. We estimate the parameters of the features probability distribution from seven expert subjects (1 SCI and 6 able-bodied) that received extensive prior training with the device, and compute the Gaussian naïve Bayes surprise of the four SCI participants with respect to the experts. The score based on all four features is compared with one based only on number of steps (an equivalent of distance walked), in terms of the separation between experts and patients that is yielded by the two indices.

## Methods

### The ReWalk™ exoskeleton

**Fig. 1:**
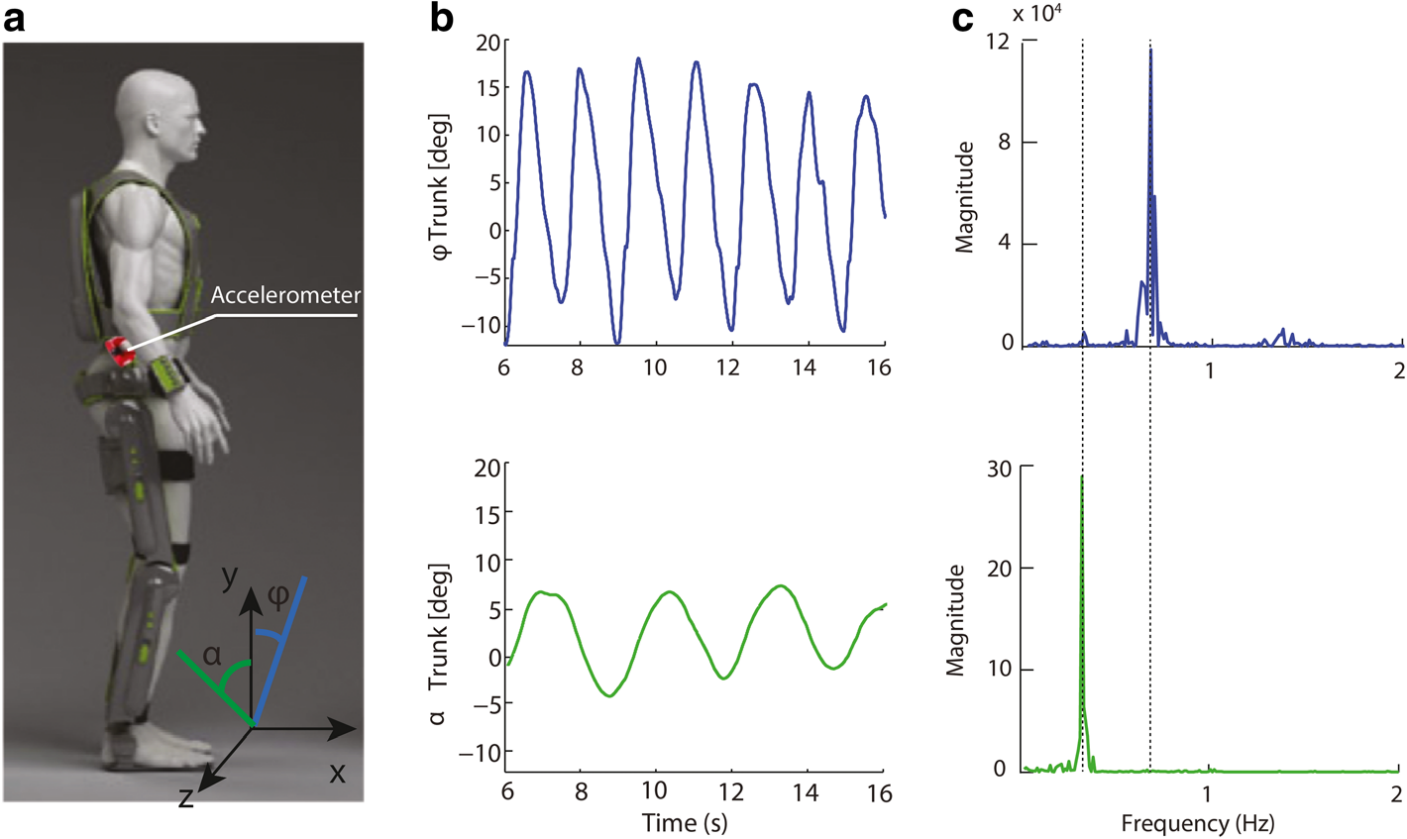
ReWalk™ exoskeleton and measured trunk angles. **a** Schematic of the ReWalk™ exoskeleton suit. The tri-axial wearable accelerometer attached on the right flank of the robot recorded the body accelerations while the subject walked with the device. Features to score walking quality were computed from the accelerations (see text). **b** The trunk angles in the frontal (x-y) and lateral (y-z) plane during walking (φ, frontal, blue line; α, lateral, green line). **c** The power spectral density plots of the trunk angles: the x-value of each maximum corresponds to the frequency of the oscillations in the plane. The step frequency corresponds to the maximum in the x-y plane

The ReWalk™ (ReWalk Robotics Inc., Marlborough, MA, USA) is a motorized lower limb exoskeleton suit designed to provide legged mobility to paraplegic patients who suffered a spinal cord injury from level T4 to L5. The device has two actuated degrees of freedom - one at the hip and one at the knee on each side – and has a passive spring-assisted dorsiflexion joint at the ankle. Figure 1a shows a schematic of the device.

The robotic suit is attached to the subject’s waist and legs by means of Velcro straps. A backpack worn by the subject contains the batteries and the controller of the device. A wristwatch connects wirelessly to the controller and is used to switch between five activity modes (sit, standup, walk, upstairs, downstairs).

Once in walking mode, the user triggers each step by leaning his/her body forward: a tilt sensor located on the left flank detects when the trunk angle exceeds a predefined threshold (8° in our case) and the robot executes a pre-programmed step. Crutches are used to stabilize the body. The robot kinematics, including the joints range of motion and stepping speed, can be personalized with the accompanying software.

### Study design

Eleven subjects participated in the study. Six able-bodied participants with no medical problems and one ASIA AIS A SCI subject (T4 injury level) constituted the pool of expert subjects (5 F, 6 M; ages: 36.9 ± 14 years). The remaining four SCI individuals were new to the device and were trained at the Rehabilitation Institute of Chicago under the supervision of a physical therapist over a period from 6 to 12 weeks. Demographic data of these subjects is reported in Table 1. All subjects were consented and Northwestern University’s Institutional Review Board approved the study.

**Table 1:** Demographic data of the four ASIA AIS A SCI participants

Subject ID	Age/Sex	Level of Injury	Time since injury	Injury cause
R09	23/M	T8	1 yr. 7 mo.	Gun-shot wound
R10	63/M	T10	7 yr. 7 mo	Ski accident
R11	33/F	T9	2 yr. 11 mo.	Gun-shot wound
R15	29/M	T10	10 mo.	Motor-cycle accident

An able-bodied expert walker was defined as one who could walk continuously for 30 min, receiving at most contact guard assistance (CGA) for safety reasons. A maximum of 3 breaks during the 30 min walking period were allowed, each resting period lasting up to a maximum of 2 min each. Subjects had to walk for at least 6 min continuously before any break could be taken. The six able-bodied subjects were trained over multiple sessions until they met this goal.

The SCI expert was a person with over 500 h of walking experience with the exoskeleton. This person has been used by ReWalk™ Robotics to act as their demo performer at numerous health conferences with the exoskeleton. The SCI expert also owns a take-home device which she uses regularly for personal mobility. Following training and a warm-up phase, subjects underwent two 6MWT spaced from each other by at least 1 min. During each 6MWT, participants walked across a 30 m straight hallway, turning at each end to reverse the direction of walking. The ReWalk™ gait settings were the same across all expert users and are reported in Table 2.

**Table 2:** ReWalk™ settings and walking speed at the beginning and end of training for all participants

Subject ID	R09		R10		R11		R15		Experts
Training session	Start	End	Start	End	Start	End	Start	End	N/A
Hip Flexion [deg]	22	22	22	22	22	22	22	22	22
Knee Flexion [deg]	31	43	31	32	45	37	34	35	35
Swing Time [ms]	1200	700	1200	600	1200	600	800	600	600
Step Delay [ms]	500	150	500	100	500	0	450	0	0
Walking speed[m/s]	0	0.178	0	0.453	0	0.355	0	0.474	0.48

The four novice participants were trained three times per week for 12 weeks, except for one patient (R10) who was trained for only 6 weeks due to an incompatibility with his work schedule. Each training session consisted of about an hour of walking with the exoskeleton, with the first 1–3 sessions focused primarily on static standing balance training and weight shifts in the ReWalk using the forearm crutches. The initial balance training sessions helps the participant learn on how to balance with the device in an upright position prior to walking initiation. Specific to the study a 6MWT was performed in the middle of each training session. The level of assistance (LOA) was kept at the minimum safe level for all patients during each 6MWT and varied depending on the skill achieved by the subject. Assistance was provided only to prevent falls and not to sustain walking and therefore should not bias the features during the test. The LOA for all subjects was either CGA or supervision at the end of training. The ReWalk™ settings changed across subjects and training sessions, and were chosen by the therapist according to the patients’ skills. At the end of training, all settings were chosen to be the closest match to the experts’ settings. The values used and the speeds achieved during a 10mWT at the beginning and end of training are reported in Table 2.

Accelerations during each 6MWT were recorded with a tri-axial wearable accelerometer (Actigraph, Florida, USA) placed on the exoskeleton in mid-sagittal position 20 cm above the hip joint, on the right side (Fig. 1a). The spot was chosen to detect the trunk motions that the subjects used to trigger the steps. Data was recorded on the device onboard memory (1 GB) at a sampling frequency of 100 Hz. Missing data from some of the patients’ sessions was due to either missed recordings or inability of the patient to complete the 6MWT.

### Walking quality features

Acceleration data corresponding to walking was visually identified and manually extracted from each recorded session. An uninterrupted period of walking in a training session is denoted as an “acceleration clip”; therefore data from each session consists of multiple clips. We excluded clips shorter than 4 s, such that each clip contains at least 400 samples.

The following 4 features (*x*_1_…*x*_4_) were computed from the acceleration clips to evaluate walking quality for each minute of walking in a 6MWT.

#### Step frequency (*x*_1_)

Each step is triggered by a motion of the trunk in the frontal (*x-y*) plane, followed by a lateral motion in the plane *y-z* (see Fig.1a), which is used to offload the swinging leg and allow clearance of the foot. These two oscillatory movements in orthogonal planes were estimated by low-pass filtering the acceleration measured in each plane with a second-order Butterworth filter (1.5 Hz cut-off frequency):

1 $$ \upvarphi ={ \tan}^{-1}\left(\frac{a_x}{a_y}\right); \kern0.5em \alpha ={ \tan}^{-1}\left(\frac{a_z}{a_y}\right) $$

with φ and *α* being, respectively, the frontal and lateral instantaneous angle. This approximation is valid when the body accelerations are small compared to gravity [22, 23].

During walking, the motions in the two planes are periodic, with the frequency of the frontal angle φ (pitch) being twice that of the lateral angle *α* (roll) (Fig. 1b). We verify this condition by computing the power spectral density (PSD) of the trunk angle in each plane (Fig. 1c). This condition ensures that the accelerations in the clip correspond to actual walking. Since one period of an oscillation in the frontal plane corresponds to a step, we estimate the step frequency *x*_1_ as:

2 $$ {x}_1= argma{x}_f\left(PSD\left(\upvarphi \right)\right),\kern0.5em \left(f>0\right) $$

where *f* is the frequency in Hz.

#### Standard deviation of the frontal angle (*x*_2_)

We hypothesized that increased training with the device will lead to smaller body motions that are reflected in a lower standard deviation of the trunk frontal angle (pitch).

3 $$ {x}_2=\sqrt{\frac{1}{M-1}{\displaystyle \sum_{t=1}^M}{\left({\upvarphi}_t-\overline{\upvarphi}\right)}^2} $$

where $\overline{\upvarphi}$ is the mean value and *M* is the number of samples in the clip.

#### Approximated energy expenditure (*x*_3_)

A quantity that correlates with energy expenditure (E) during walking was computed by summing the absolute values of the accelerations and integrating them over a time interval (epoch), after gravity was removed with a high-pass filter (Butterworth 2^nd^ order, cut-off frequency 0.25 Hz).

4 $$ {E}_k={\displaystyle {\int}_{t_k}^{t_{k+1}}\left(\left|{a}_x(t)\right|+\left|{a}_y(t)\right|+\left|{a}_z(t)\right|\right)}dt $$

where the subscript k indicates the k-th epoch and the epoch length ([*t*_*k*_, *t*_*k* + 1_]) is set to 0.5 s. The unit of measurement of E is commonly known as “acceleration counts” or “counts” and it measures the movement intensity over the 3 axes. Although the correlation with energy expenditure has only been verified in free walking subjects [24], we believe this is still a reasonable assumption in our setup.

Counts are normalized by the number of steps taken by the subject in the acceleration clip, yielding the energy expenditure (counts) per step:

5 $$ {x}_3=\frac{{\displaystyle {\sum}_k}{E}_k}{N_{steps}} $$

#### Number of steps (*x*_4_)

Number of steps is used in place of distance walked to ensure that the feature is independent of the step length, which varies amongst subjects. For each walking clip in a given session, the number of steps was computed from the step frequency and the duration of the clip *T*_*c*_. This feature characterizes the ability of taking multiple consecutive steps.

6 $$ {x}_4={x}_{1,c}{T}_c $$

where the subscript *c* refers to the indexed clip.

We compute a feature vector ***x*** = (*x*_1_, …, *x*_4_)^*T*^ for every minute of a 6MWT; if multiple clips (walking bouts) are present within the minute window, the values of the features are averaged across clips, with the exception of number of steps, which was summed.

### Gaussian naïve Bayes surprise

Our goal is to score a patient’s skill at walking with the exoskeleton relative to a trained expert user, according to the values of the features. We start by estimating the probability distribution of the features across the expert subjects and then measure the likelihood of a patient being an expert user.

A simple yet effective approach in many cases is to assume that the features are conditionally independent, which is also known as the naïve Bayes assumption [25]. If the features are normally distributed, the joint probability distribution of the features over the expert subjects is:

7 $$ p\left({x}_1,\dots, {x}_4\Big|{\mu}_{i,H},{\sigma}_{i,H}^2\right)={\displaystyle \prod_{i=1}^4}p\left({x}_i\Big|{\mu}_{i,H},{\sigma}_{i,H}^2\right)={\displaystyle \prod_{i=1}^4}\frac{1}{\sqrt{2\pi {\sigma}_{i,H}^2}}{e}^{\frac{-{\left({x}_i-{\mu}_{i,H}\right)}^2}{2{\sigma}_{i,H}^2}} $$

where *x*_*i*_ indicates the *i*-th feature value for a subject, and *μ*_*i*,*H*_ and *σ*_*i*,*H*_ are, respectively, the mean and standard deviation of that feature across the expert subjects.

 By taking the negative natural logarithm of (7) we obtain the Gaussian naïve Bayes surprise Ψ:

8 $$ \Psi =- \ln p=-{\displaystyle \sum_{i=1}^4}\left(\frac{1}{2} \ln \left(2\pi {\sigma}_{i,H}^2\right)+\frac{{\left({x}_i-{\mu}_{i,H}\right)}^2}{2{\sigma}_{i,H}^2}\right) $$

We can interpret (8) as the log probability of a subject being expert, under the assumption of independence of the features. In our model, the farther the feature values of a subject from the mean values of expert subjects, the larger the surprise. Importantly, each feature in (8) is weighted by the inverse of its variance: thus, features with lower variance across expert subjects are weighed more, as they are considered more reliable for quantifying walking skills.

In order to normalize the values of Ψ, we compute the z-score of the Gaussian naïve Bayes surprise with respect to its distribution across the expert subjects Ψ_*h*_:

9 $$ {Z}_{\Psi}=\frac{\Psi -{\overline{\Psi}}_h}{\sigma_{\Psi_h}} $$

where Ψ is the Gaussian naïve Bayes surprise for a given subject, and ${\overline{\Psi}}_h$ and ${\sigma}_{\Psi_h}$ are, respectively, the mean and the standard deviation of the distribution Ψ_*h*_ (i.e. the surprise across expert subjects). This distribution is estimated by a leave-one-subject-out cross validation, such that each expert subject is scored relative to all the others. Thus, the z-score for the *j*-th expert subject is:

10 $$ {Z}_{\Psi_{h,j}}=\frac{\Psi_j-{\overline{\Psi}}_{h\backslash \left\{j\right\}}}{\sigma_{\Psi_{h\backslash \left\{j\right\}}}}\kern1.75em \left(j=1,\dots, S\right) $$

where *S* is the number of expert subjects and the notation *h*\{*j*} indicates that the value is computed over all the subjects except subject *j*.

The z-score *Z*_Ψ_ (9) indicates by how many standard deviations a subject’s performance is above or below the mean performance of the experts. If a subject’s z-score is within ±2, we consider the subject’s walking skills are not significantly different from that of an expert user.

We evaluate the discriminatory power of the index at separating new users from experts by comparing the score values when *Z*_Ψ_ is computed using all the features with the case where only one feature (i.e. distance walked or, equivalently, number of steps) is used. The latter is chosen since its analogue (i.e. distance walked) is amongst the most common clinical outcome measures currently used with robotic exoskeletons to quantify expertise or ability. In this case the Gaussian naïve Bayes surprise (8) reduces to:

11 $$ \Psi =\frac{1}{2} \log \left(2\pi {\sigma}_4^2\right)+\frac{{\left({x}_4-{\mu}_4\right)}^2}{2{\sigma}_4^2} $$

## Results

### Expert subjects’ scores

**Fig. 2:**
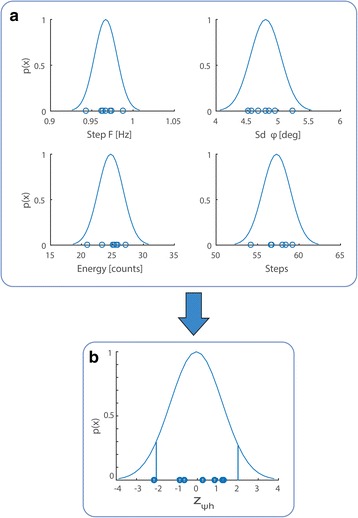
Feature distributions and z-scores across expert subjects. **a**Gaussian probability distributions of the features fitted to the data from expert subjects. Each dot is one subject. Probability values are normalized to 1. **b** All features are combined into a single score and each expert subject is scored relative to all other experts. The resulting z-score represents how many standard deviations a subject is from the experts mean performance. Z-scores for expert subjects are all within ±2 except for one subject (see text) indicating that their performances were similar. If a patient’s score lies within this range, the patient is considered equivalent to an expert user

Probability distributions of features were computed by fitting Gaussians to the walking data of expert subjects (Fig. 2a). Mean step frequency and number of steps across experts were 0.97 ± 0.014 Hz and 57.3 ± 1.67 steps, respectively (mean ± standard deviation). Mean standard deviation of trunk frontal tilt and counts (approximated energy expenditure) were 4.8° ± 0.24° and 24.7 ± 2.02 counts, respectively. These values are the average across each minute of the 6MWT for all subjects. Step frequency was the feature with the lowest standard deviation (0.014 Hz) across subjects. These distributions characterize successful control behavior.

In order to combine the individual features into a joint score, the degree to which the subject’s behavior differs from that of the expert population is quantified for each feature. From the Gaussian fit, we can read out the probability and, according to naïve Bayes, we then multiply these probabilities across all features. Each expert subject was scored relative to the others by computing the z-score of the Gaussian naïve Bayes surprise (10): individual scores were all within ±2, except for one able-bodied subject who had a score of -2.2 because the robot malfunctioned and stopped twice during her test, thus affecting her total number of steps. The mean z-score across experts was -0.04 (Fig. 2b). Importantly, the SCI expert did not score differently from the able-bodied: therefore, her features fit the distribution of the able-bodied subjects, thus confirming that performance was comparable and consistent across all expert subjects. This result indicates that the features characterizing expert performance are not significantly different across experts and therefore our pool of subjects is sufficiently uniform. We thus have a measure that quantifies deviation from typical expert performer level.

### Patients’ scores during training

**Fig. 3:**
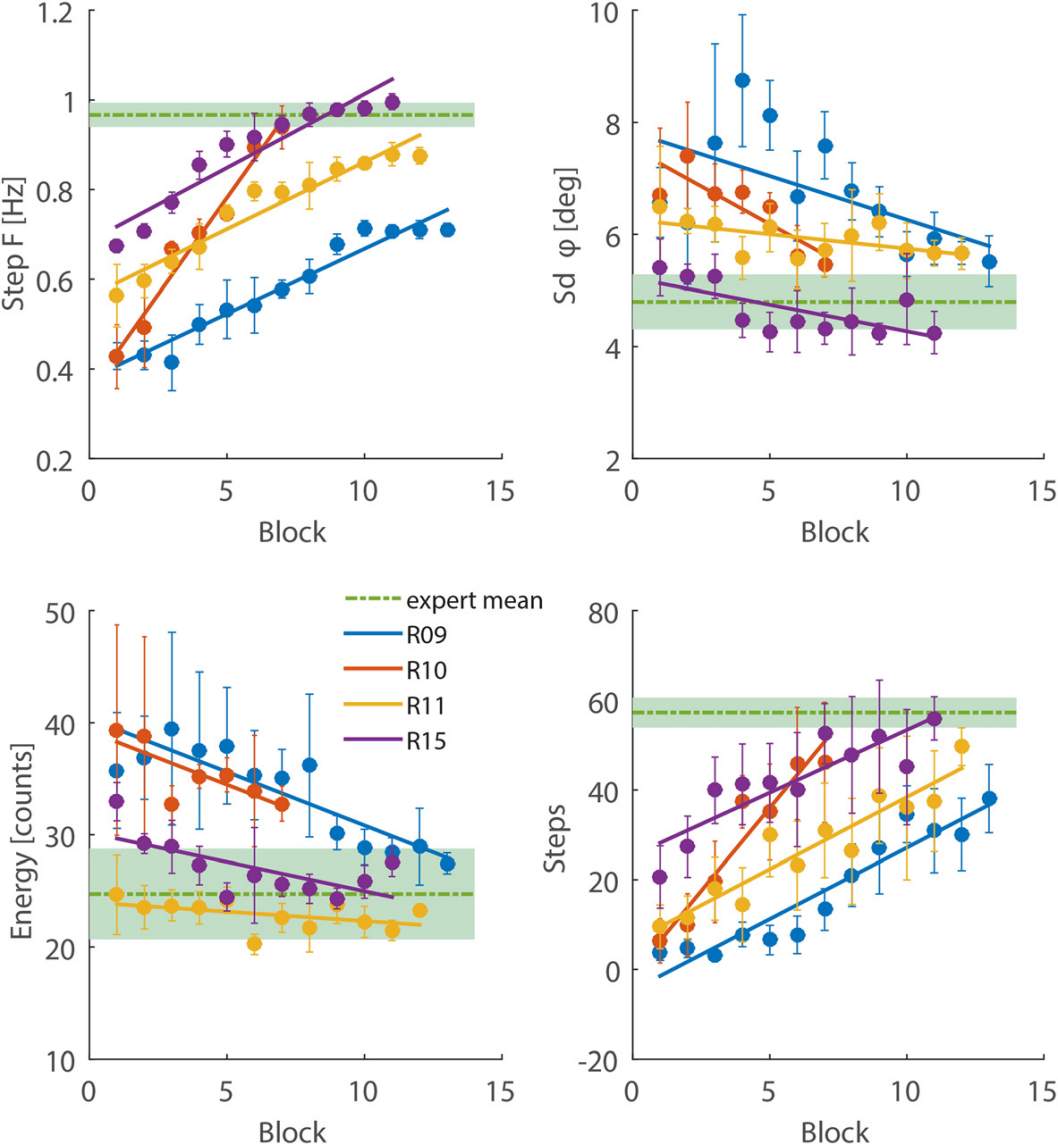
Improvements across training for individual patients. Each plot displays an individual feature and each color denotes a different patient. Data is averaged over 2 sessions (1 block). Solid lines are the least squares linear fit to the data. The green dotted line and the shaded areas indicate mean ±2 standard deviations across expert subjects. Error bars are ±1 standard deviation from the mean

Improvements in patients were reflected in the features values getting closer to the mean values of expert subjects (Fig. 3). Step frequency (*x*_1_) and number of steps (*x*_4_) were positively correlated with session for all patients (Pearson’s *r* > 0.87, *p* < 0.001); standard deviation of the trunk angle *φ* (*x*_2_) and energy expenditure (*x*_3_) decreased over time for all patients (Pearson’s *r* < − 0.6, *p* < 0.05), except for patient R11, whose acceleration counts (energy) were about the same than that of the experts for the entire training. Learning rates and initial values of each feature varied drastically across patients, confirming that different features captured different aspects of learning.

**Fig. 4:**
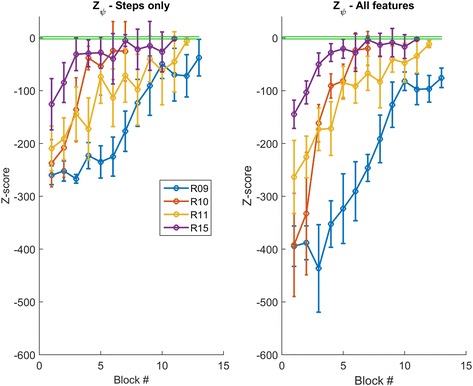
Patient z-scores during training. Patients’ improvements across training when (left) steps are used as the only feature or (right) all four features are combined to compute the z-score. Data is mean z-score across 2 sessions. The green lines indicate ± 2 standard deviations from the mean z-score of the expert subjects. Patients’ separation increases when the combined z-score is used. Error bars are one standard deviation from the mean

We compared the z-scores when using only steps as feature with that obtained when all the features are used. Both scores showed that patients improved as a result of training (Fig. 4). Using all the features magnified separation across patients as well as from experts, and overall reduced the standard deviation of the score within sessions, thus aiding detection of improvements towards optimal expertise.

**Fig. 5:**
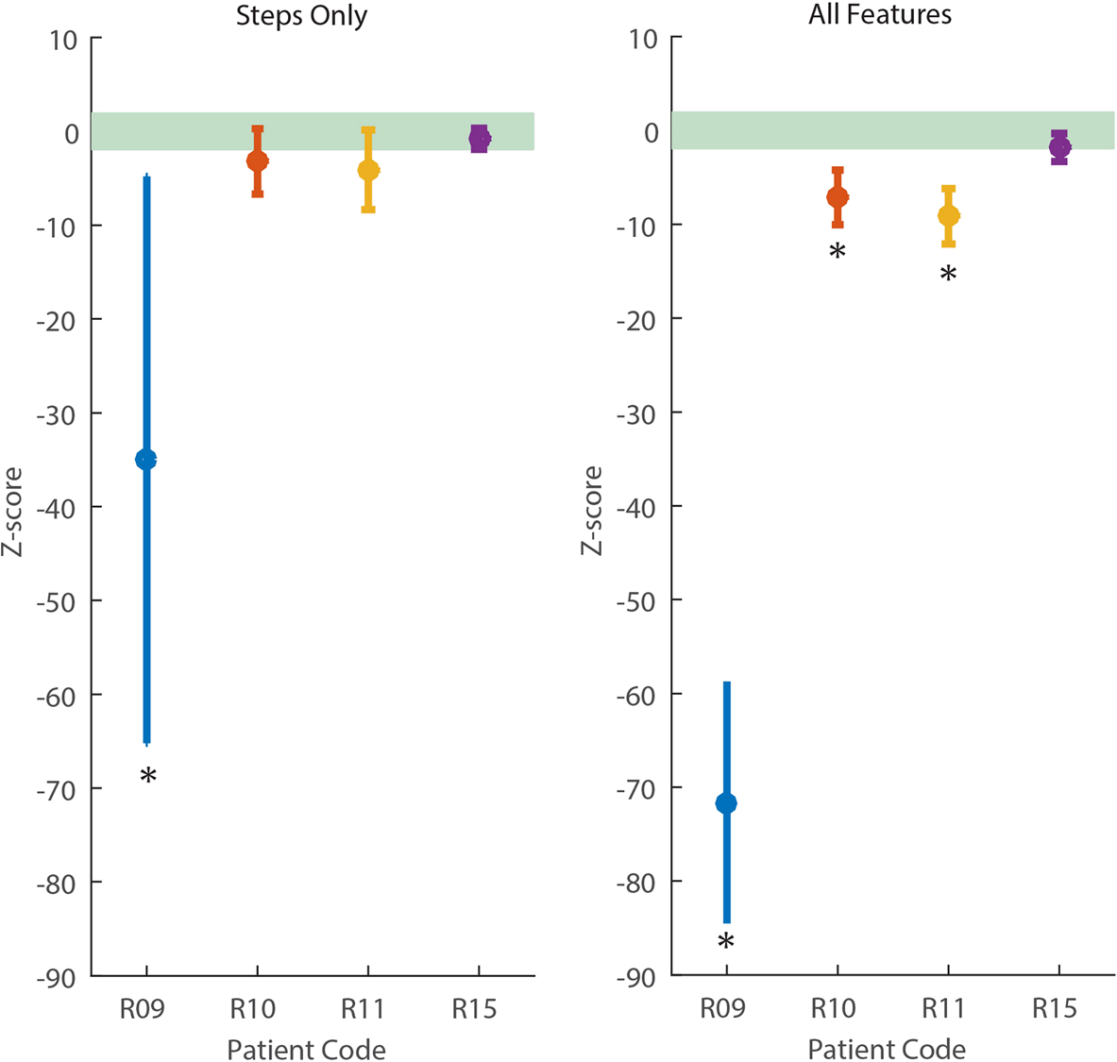
Patient scores at the end of training. Using multiple features (right) improves patients’ discriminability from the experts (green shaded area indicates ± 2 standard deviations from the mean experts’ score) as compared to using steps as the only feature (left). Data is mean z-score over 2 sessions and error bars are 1 standard deviation from the mean. Asterisk (*) indicates the score is significantly below –2 (Wilcoxon signed rank test, *p* < 0.001), i.e. the patient has not reached the experts’ level

We compare the two scores in terms of their discriminatory power, by testing which patients have a z-score that is above -2 standard deviations from the experts in the last two training sessions. Using the score based on steps yielded that 3 patients (R10, R11 and R15) were not significantly different from the experts (One tailed Wilcoxon signed rank test, *p* > 0.13). In contrast, when all features are used, only patient R15 is classified as expert (*p* = 0.42), with the others all being scored as non-experts (*p* < 0.001) (Fig. 5). The result of the combined features model was in agreement with the evaluation of the physical therapist that trained the patients and evaluated their skills independently from the model prediction.

## Discussion

In this study, a simple setup consisting of a wearable accelerometer was used to quantify walking skills in subjects with spinal cord injury who were trained to walk with an exoskeleton. A set of features was computed from the trunk accelerations of the subjects as they were trained to use the ReWalk™. Features were combined into a single index of walking quality, the Gaussian naïve Bayes surprise, which scored patients relative to expert subjects, who received extensive prior training with the exoskeleton. We found that our index has a higher discriminatory power than the index that used number of steps as the only feature. A discriminatory score with increased sensitivity can better support clinicians’ decisions on whether a participant needs more training or is ready to use the robotic exoskeleton at home. This is crucial, given that an individual with complete SCI and walking with a 50 lb powered exoskeleton using a walker or forearm crutches is in a precarious situation, and significant expertise is required to avoid adverse events while moving in a community environment, even in the presence of a companion.

Previous studies trying to assess how SCI individuals perform with lower limb exoskeletons have used a combination of standard clinical outcome measures and other specialized analysis, including kinematics, dynamics and physiological features [14–20]. Despite using multiple features to characterize walking skills with a particular device, these previous studies have looked at each variable individually. It is thus unclear which features are more important to evaluate the performance of a subject for independent walking. In our combined score model, each feature is weighted by the inverse of its variance, as estimated from a pool of expert users. Therefore, our method automatically infers the feature importance from the experts’ data, in order to evaluate the walking skills of a new subject.

Furthermore, previous exoskeleton research measured performance at a single time point, usually following training. In this work the change in individual features across training sessions was tracked, which allows analyzing the learning curve of participants. This information could help clinicians at predicting patients‘ expertise and determine how much additional training is needed to reach expert proficiency. Furthermore, given the early stage of development of robotic exoskeletons, additional insights into the patients’ learning curve could guide the design of future exoskeletons and the planning of training sessions, by considering how each feature changes for different patients.

Our work has several limitations: considering our sample size, a study with a larger pool of subjects is crucial to validate the results and remains as future work. Secondly, only 4 features from the trunk accelerations to evaluate walking quality were computed; incorporating other features could capture additional factors that affect walking skills with the exoskeleton. Similarly, deriving features that directly quantify safety, such as walking stability or probability of falls, could provide a significant improvement over the current model. Interestingly, other studies have tried to infer the stability of walking from body accelerations in both healthy and subjects at risk of falls [26–28]; however all these studies were performed on freely walking subjects and how to translate these methods to subjects walking with an exoskeleton should be investigated.

Ideally the features should be independent, which is not true in our case, as the standard deviation of the trunk tilt and the acceleration counts are expected to be strongly correlated. The feature independence (naïve Bayes) assumption was chosen to keep the model simple and to prevent overfitting of the probability distribution parameters. This assumption could be relaxed if data from more subjects are available. On the other hand, the naïve Bayes assumption proved to work well in several problems even where the features are actually not independent [29]. Similarly, it has been shown that redundant features can still describe different aspects of walking skills during training in individuals with spinal cord injury [30].

## Conclusion

The current study provides a first step towards the construction of an index that quantifies walking skills in exoskeletons, by integrating multiple features (metrics) in a principled way. Combining multiple features could help clinicians to determine when a patient has reached a level of ability to walk safely at home and in the community with a robotic exoskeleton. We used a simple sensor to infer relevant quantitative data about walking skills in individuals with complete SCI and were able to track their progress over time. More advanced wearable sensors [31, 32] could be used to derive additional features, as well as to provide feedback on the optimality of the subject movements and potentially facilitate learning. Future work is necessary to determine the most effective feedback system to address this question. Overall, the proposed approach of constructing an index of walking performance could be applied to any other situation where expert performance is measured along with existing performance. This could be useful across a wide range of rehabilitation studies and beyond.

## Abbreviations

6MWT: 6 minute walk test
10mWT: 10 meter walk test
CGA: contact guard assistance
LOA: level of assistance
SCI: spinal cord injury
